# Visual and refractive outcomes after implantation of two models of trifocal intraocular lenses in eyes with previous corneal ablation to treat hyperopia

**DOI:** 10.1186/s40662-023-00366-x

**Published:** 2023-12-07

**Authors:** Fernando Mayordomo-Cerdá, Julio Ortega-Usobiaga, Julio Baviera-Sabater, Rafael Bilbao-Calabuig, Fernando Llovet-Osuna, Vasyl Druchkiv, Rosario Cobo-Soriano

**Affiliations:** 1 Clinica Baviera-AIER Eye Group, GV Marqués del Turia 9, 46005 Valencia, Spain; 2 Clinica Baviera-AIER Eye Group, Leizaola Lehendakariaen Kalea, 48011 Bilbao, Spain; 3 Clinica Baviera-AIER Eye Group, Pº Castellana 20, 28046 Madrid, Spain; 4 College of Medicine, Cardenal Herrera-CEU University, Valencia, Spain; 5 Department of Research and Development, Clinica Baviera-AIER Eye Group, Valencia, Spain; 6 Department of Ophthalmology, UKE—University Medical Center Hamburg-Eppendorf, Hamburg, Germany; 7 Universidad Francisco de Vitoria, Madrid, Spain; 8 Clinica Baviera-AIER Eye Group, Calle La Merced 12, 4, 10, 46300 Utiel, Valencia, Spain

**Keywords:** Trifocal intraocular lens, Laser corneal ablation, Hyperopia, Spherical aberrations

## Abstract

**Background:**

To assess whether a trifocal intraocular lens (IOL) with neutral spherical aberration (SA) provides better visual and refractive outcomes than a trifocal IOL with negative SA after hyperopic corneal laser ablation.

**Methods:**

This is a retrospective comparative study. Patients were classified according to the IOL implanted after cataract or clear lens phacoemulsification [group 1, PhysIOL FineVision Pod-F (negative SA); group 2, Rayner RayOne Trifocal (neutral SA)]. We evaluated uncorrected distance visual acuity (UDVA), corrected distance visual acuity (CDVA), uncorrected intermediate visual acuity (UIVA), uncorrected near visual acuity (UNVA), predictability, safety, efficacy, and satisfaction.

**Results:**

198 eyes of 119 patients met the inclusion criteria. Group 1 comprised 120 eyes and group 2 comprised 78 eyes. At completion, the refractive and predictability results were significantly better in group 1 than in group 2 for manifest refraction spherical equivalent (MRSE) (*P* < 0.001). Differences were not significant for UDVA (*P* = 0.647), CDVA (*P* = 0.343), UIVA (*P* = 0.059), UNVA (*P* = 0.382), binocular UIVA (*P* = 0.157), or binocular UNVA (*P* = 0.527). Safety and efficacy indices in refractive lens exchange (RLE) eyes were 0.96 and 0.91, and 0.89 and 0.93 in groups 1 and 2, respectively (*P* = 0.254 and 0.168). Patient satisfaction was similar in both groups (*P* > 0.05, all items).

**Conclusion:**

In eyes previously treated with hyperopic corneal ablation, implantation of a trifocal IOL with neutral SA provided better efficacy and safety outcomes but worse predictability outcomes than those obtained with a trifocal model with negative SA.

## Background

In the last 30 years, laser corneal refractive surgery (LCRS) has been the most widely used surgical procedure for the correction of refractive errors in nonpresbyopic patients [1]. As those patients grew older, the number with presbyopia or cataract requesting a new refractive procedure increased. Lens phacoemulsification with multifocal intraocular lens (IOL) implantation is the most effective technique for achieving spectacle independence in presbyopic or cataractous patients [2–4]. Consequently, this procedure is becoming more common in patients with prior LCRS who wish to be independent of glasses or contact lenses [5–8].

Excimer laser corneal ablation induces a positive or negative shift in corneal spherical aberration (SA) values after myopic and hyperopic ablation, respectively [9, 10]. Most currently available trifocal IOLs have negative SA to compensate for the natural positive SA of the human cornea, which could be appropriate for a patient with previous myopic LCRS, but not for a patient who underwent hyperopic LCRS with postoperative corneal negative SA. It is important to consider this when selecting a trifocal IOL to counteract the corneal changes induced by photoablation. An IOL with a positive or a neutral SA should provide better visual outcomes after hyperopic ablation. However, to our knowledge, this hypothesis has not been tested. The present study was designed to determine whether an aspherical trifocal IOL with a neutral SA provides better visual and refractive outcomes and patient satisfaction than an aspherical trifocal IOL with a negative SA in eyes treated with hyperopic LCRS.

## Methods

### Design

This multicenter, multi-surgeon, single-protocol, retrospective, case series study consecutively enrolled eyes that had undergone lensectomy with implantation of a trifocal IOL after a previous LCRS to treat hyperopia at our institution (all preoperative data known). To provide surgeons with significant information about outcomes, we separated and compared the results between the two groups, depending on the type of IOL implanted: group 1 included patients who received an aspheric trifocal IOL with a negative SA, and group 2 included patients who received an aspheric trifocal IOL with a neutral SA.

### Subjects

Data were recorded using the central computerized clinical records system in Clinica Baviera, Spain. The study period was from 1999-11-23 (first visit before LCRS; YYYY-MM-DD) to 2021-09-30 (last available postoperative visit). Laser treatments were performed between 1999-11-23 and 2017-05-15. Lens surgeries were performed between 2016-09-19 and 2021-08-11. We have included patients who have undergone cataract surgery (no greater than NO1/NC1, C1, in LOCS III scale) or refractive lens exchange (RLE). The term “lensectomy” in the text is used to refer to both the cataract and RLE groups and therefore includes both types of phacoemulsification.

The study consists of a research on existing data. These data were recorded anonymously using identifiers linked to the subjects. Given the retrospective nature of the study, it was approved by our institutional legal and ethical committee with an exempt review (Ethical Committee of Clinica Baviera, Spain). All patients received detailed information before surgery and provided written informed consent for multifocal lensectomy after LCRS. They also provided informed consent for the use of anonymous and aggregated medical data for the study.

The study inclusion criteria were as follows: (1) lens surgery [RLE or cataract (NO1/NC1, C1) with implantation of a trifocal IOL in eyes previously treated with laser-assisted in situ keratomileusis (LASIK)] for correction of hyperopia, (2) potential visual acuity [baseline pre-LCRS logMAR corrected distance visual acuity (CDVA) < 0.5], (3) at least three months of follow-up after implantation, and (4) no corneal laser enhancement. The exclusion criteria were: (1) eyes with subnormal optics, such as corneal topographic abnormalities (small optical zones, decentered ablations, suspected ectasia), and (2) any baseline anatomical disorder (vitreoretinal or surface/anterior segment disorder) or any perioperative anatomical complications (corneal and/or lens surgeries) to rule out organic disease that could mask the functional outcomes of both refractive procedures.

### Intraocular lenses

The diffractive trifocal IOLs implanted during the study period were FineVision Pod-F (PhysIOL, Liège, Belgium) in group 1 and RayOne Trifocal (Rayner, Worthing, United Kingdom) in group 2. Both IOLs were manufactured using foldable hydrophilic acrylic materials. The FineVision Pod-F (single-piece, double-C loop haptics) combines two diffractive structures adjusted to offer a + 3.50 D addition for near vision and a + 1.75 D addition for intermediate vision; it has an aspheric profile with − 0.11 µm of SA. The RayOne Trifocal IOL provides a neutral aspheric optical profile, that is, 0 µm of SA, with 16 diffractive steps, a + 3.50 D near addition, and a + 1.75 D intermediate addition. The pupil size for SA values of both lenses (RayOne and FineVision) is 6 mm.

### Surgical procedures

The corneal and lens procedures were performed by experienced surgeons using homogeneous perioperative protocols. The LCRS procedure was LASIK in all cases and was performed using two microkeratomes with nasal hinges (Moria LSK-ONE and Moria ONE-USE-PLUS-SBK, Microtech Inc., Moria Ophthalmic Instruments, Anthony, France) and three excimer laser models [Technolas 217C, 217-Z-100 (Bausch & Lomb, Claremont, California, USA), Mel-80 (Carl Zeiss Meditec, Jena, Germany), and WaveLight-Allegretto Wave-Eye-Q (Alcon Laboratories, Fort Worth, Texas, USA)]. All LCRS data are available.

Patients who had previously undergone LASIK ablation to treat hyperopia returned to the clinic for lens surgery because of reduced distance and/or near visual acuity caused by presbyopia and/or cataracts. After selection of an appropriate lens, standard, uneventful phacoemulsification was performed with implantation of a trifocal IOL in the capsular bag.

The online American Society of Cataract and Refractive Surgery (ASCRS) calculator (https://iolcalc.ascrs.org) and/or the Barret True-K formula (https://www.apacrs.org/apacrsbiometry/True-K.aspx) were used for IOL calculation by entering refractive, keratometric, topographic, and biometric data based on a multiformula approach. We aimed for emmetropia, so we selected the average lens power in all cases.

### Clinical evaluation

All surgical procedures were performed at our institution, using homogeneous preoperative assessment protocols. Patients underwent a complete ophthalmologic examination that included the measurement of visual acuity data, namely, Snellen distance visual acuity (Snellen auto chart projectors, Topcon Corp, Tokyo, Japan), Jaeger near and intermediate visual acuity (Runge Near Vision Card, Good-Lite, Elgin, Illinois, USA), and refraction (uncorrected and corrected, manifest, and cycloplegic). Refractive status, registered by an optometrist, included uncorrected distance visual acuity (UDVA), CDVA, uncorrected intermediate visual acuity (UIVA) at 80 cm, and uncorrected near visual acuity (UNVA) at 40 cm (visual acuities were tested under photopic conditions, at approximately 85 cd/m^2^). The patients also underwent topography, slit lamp biomicroscopy, ocular surface/tear film evaluation, and fundoscopy.

However, owing to the diversity in practice locations, study time points, and development of devices over time, preoperative topographic evaluation was not standardized. The three corneal topographers used during the study period were the Orbscan II (Bausch&Lomb, Claremont, California, USA), Pentacam (Oculus Optikgerate GmbH, Wetzlar, Germany), and Wavelight-Oculyzer (Alcon Laboratories, Foxworth, Texas, USA). Nevertheless, to assess the impact of previous corneal hyperopic ablation and to avoid bias when evaluating our results, the preoperative corneal Z4(0) and high-order aberration (HOA) values obtained on Pentacam were also studied and compared.

Preoperative examination for lens surgery also included endothelial cell count (SP 3000P; Topcon, Capelle, The Netherlands) and macular optical coherence tomography (SOCT Copernicus-REVO, Optopol-Tech, Zawircie, Poland). Biometric parameters were assessed using an optical biometer (IOLMaster 500; Carl-Zeiss-Meditec AG).

### Refractive and visual measures

The main measurements were visual and refractive outcomes and patient satisfaction, which were obtained from the last available visit, with at least three months of follow-up after implantation. Visual outcomes included average logMAR UDVA, CDVA, UIVA and UNVA. Refractive data included postoperative sphere, cylinder, manifest refraction spherical equivalent (MRSE), and accuracy (percentage of eyes within ± 0.50 D and ± 1.00 D). Safety outcomes were defined as the percentage of eyes with a loss of ≥ 1 or ≥ 2 lines of CDVA between the time after LCRS and lens surgery; the lines presented in the graphs correspond to a change of 0.1 on logMAR scale. For instance, a patient with preoperative CDVA 0 logMAR (decimal 1.0) and postoperative UDVA 0.1 logMAR (decimal 0.8) would fall in “1 worse” on the efficacy Bar-Chart.

Efficacy outcomes were measured as the percentage of eyes with a difference between post-LCRS CDVA and post-lensectomy UDVA ≥ 0 lines. The safety index is defined as the ratio of mean preoperative CDVA to mean postoperative CDVA; and the efficacy index as mean postoperative UDVA to mean preoperative CDVA. Although the degree of cataract was not advanced enough to induce significant changes in CDVA, we have calculated Safety and Efficacy indices for the RLE group only.

A patient satisfaction questionnaire was used by our group in previous studies [4, 7].

### Statistical analysis

When comparing independent groups, the distributions were assessed for outliers, normality, and homogeneity of variance. The outliers were assessed using the box plot method. Normality was assessed using the Shapiro–Wilk test and quantile-to-quantile (Q–Q) plots. Homogeneity of variances was verified using the Levene’s test. In most cases, these assumptions were met, and an independent *t*-test was performed. In case where the assumptions of the parametric test were not satisfied, non-parametric Mann–Whitney test was performed, and medians and quartiles were reported. Otherwise, we report usual means and standard deviations with differences tested with *t*-test.

## Results

The study sample comprised 198 eyes (97 right, 49.0%) of 119 patients (73 females, 61.3%) who underwent primary hyperopic LCRS and subsequent implantation of a trifocal IOL at our institution. The patients were followed up for at least three months after lensectomy. The series was divided into two groups according to the type of trifocal IOL implanted: group 1 (FineVision IOL, n = 120 eyes) and group 2 (RayOne IOL, n = 78 eyes). Binocular implantation was performed in 46 patients in group 1 and 33 patients in group 2. The mean time from LCRS to lensectomy surgery was 11.79 ± 3.47 years, with a range of 1.6 to 20.5 years. The mean time from lensectomy to the last visit was 18.09 ± 16.53 months (range, 0.2 to 56.4 months). Table 1 displays the additional demographic data and Table 2 shows the preoperative and postoperative visual and refractive data before and after the LCRS for both groups.

**Table 1:** Demographics and study data

Parameter	Group 1 (n = 120)	Group 2 (n = 78)	Total (n = 198)	*P* value
Eye				0.951^a^
OD	59 (49.2%)	38 (48.7%)	97 (49.0%)	
OS	61 (50.8%)	40 (51.3%)	101 (51.0%)	
Treatment laterality				0.211^a^
Monocular	28 (37.8%)	12 (26.7%)	40 (33.6%)	
Binocular	46 (62.2%)	33 (73.3%)	79 (66.4%)	
Gender				0.312^a^
Female	48 (64.9%)	25 (55.6%)	73 (61.3%)	
Male	26 (35.1%)	20 (44.4%)	46 (38.7%)	
Age (years)				0.897^b^
Mean (SD)	58.6 (8.9)	58.8 (7.8)	58.6 (8.5)	
Primary treatment type				0.159^a^
PRK	3 (2.5%)	0 (0.0%)	3 (1.5%)	
LASIK	117 (97.5%)	78 (100.0%)	195 (98.5%)	
Primary laser				0.316^a^
ALLEGRETTO	4 (3.3%)	0 (0.0%)	4 (2.0%)	
MEL-80	31 (25.8%)	18 (23.1%)	49 (24.8%)	
TECHNOLAS	85 (70.8%)	60 (76.9%)	145 (72.7%)	
Phaco type				0.002^a^
Cataract	65 (54.2%)	25 (32.1%)	90 (45.5%)	
Lensectomy	55 (45.8%)	53 (67.9%)	108 (54.5%)	

*SD* = standard deviation; *PRK* = photorefractive keratectomy; *LASIK* = laser in situ keratomileusis

^a^Chi-squared test

^b^*t*-test

**Table 2:** Laser corneal refractive surgery (LCRS). Changes in refractive and visual data for group 1 (FineVision) *vs.* group 2 (RayOne): pre-LCRS, LCRS, and post-LCRS

Parameter	Group 1 (n = 120)	Group 2 (n = 78)	*P* value
Pre-LCRS			
* Sphere (D)*			
Range	0.00 to 6.50	0.00 to 5.50	
* Cylinder (D)*			
Range	− 6.75 to 0.00	− 5.00 to 0.00	
* MRSE (D)*			
Range	− 2.25 to 5.25	− 2.00 to 4.50	
Mean (SD)	1.93 (± 1.38)	2.02 (± 1.27)	0.672^b^
* J0 (D)*			
N	67	53	
Range	− 2.25 to 2.59	− 0.62 to 1.92	
Median (Q25/Q75)	− 0.09 (− 0.32/0.31)	− 0.02 (− 0.31/0.32)	0.956^a^
* J45 (D)*			
Range	− 2.17 to 1.92	− 1.08 to 1.61	
Median (Q25/Q75)	0.00 (− 0.17/0.16)	0.00 (− 0.09/0.12)	0.283^a^
* UDVA (logMAR)*			
Range	0.00 to 1.70	0.00 to 1.70	
Median (Q25/Q75)	0.52 (0.22/0.87)	0.40 (0.15/0.52)	**0.001**^**a**^
* CDVA (logMAR)*			
Range	− 0.08 to 0.30	0.00 to 0.40	
Median (Q25/Q75)	0.00 (0.00/0.05)	0.01 (0.00/0.05)	**0.021**^**a**^
LCRS treatment			
* Sphere (D)*			
Range	1.00 to 7.50	1.00 to 6.00	
* Cylinder (D)*			
Range	− 6.75 to 0.00	− 5.75 to 0.00	
* MRSE (D)*			
Range	− 0.75 to 6.25	− 0.38 to 5.38	
Mean (SD)	2.52 (± 1.22)	2.69 (± 1.33)	0.376^b^
* J0 (D)*			
Range	− 1.85 to 2.59	− 0.62 to 1.92	
Median (Q25/Q75)	0.00 (− 0.20/0.33)	0.00 (− 0.20/0.25)	0.805^a^
* J45 (D)*			
Range	− 2.17 to 1.92	− 0.97 to 2.49	
Median (Q25/Q75)	0.00 (− 0.13/0.11)	0.00 (− 0.04/0.11)	0.148^a^
Post-LCRS			
* Sphere (D)*			
Range	− 1.75 to 2.00	− 2.00 to 2.25	
* Cylinder (D)*			
Range	− 2.00 to 0.00	− 2.00 to 0.00	
* MRSE (D)*			
Range	− 2.00 to 1.75	− 2.25 to 1.50	
Mean (SD)	− 0.22 (± 0.75)	− 0.27 (± 0.78)	0.681^b^
* J0 (D)*			
Range	− 0.50 to 0.98	− 0.47 to 0.87	
Median (Q25/Q75)	0.09 (0.00/0.25)	0.04 (0.00/0.32)	0.574^a^
* J45 (D)*			
Range	− 0.82 to 0.49	− 0.43 to 0.50	
Median (Q25/Q75)	− 0.09 (− 0.21/0.00)	0.00 (− 0.10/0.09)	**0.019**^**a**^
* UDVA (logMAR)*			
Range	− 0.10 to 1.00	0.00 to 0.70	
Median (Q25/Q75)	0.09 (0.01/0.16)	0.10 (0.02/0.22)	0.391^a^
* CDVA (logMAR)*			
Range	− 0.08 to 0.15	0.00 to 0.26	
Median (Q25/Q75)	0.01 (0.00/0.05)	0.05 (0.00/0.10)	**0.023**^**a**^

*n* = number of available cases; *D* = diopters; *MRSE* = manifest refraction spherical equivalent; *SD* = standard deviation; *UDVA* = uncorrected distance visual acuity; *CDVA* = corrected distance visual acuity

^a^Mann–Whitney test

^b^*t*-Test

Bold values indicate statistically significant

Regarding pre-lensectomy corneal SA, in the FineVision group the mean Z4(0) was − 0.19 ± 0.21 µm and in the RayOne group − 0.17 ± 0.29 µm (*P* = 0.713). HOA in the FineVision group was 0.25 ± 0.12 µm and in the RayOne group 0.22 ± 0.10 µm (*P* = 0.062).

**Fig. 1:**
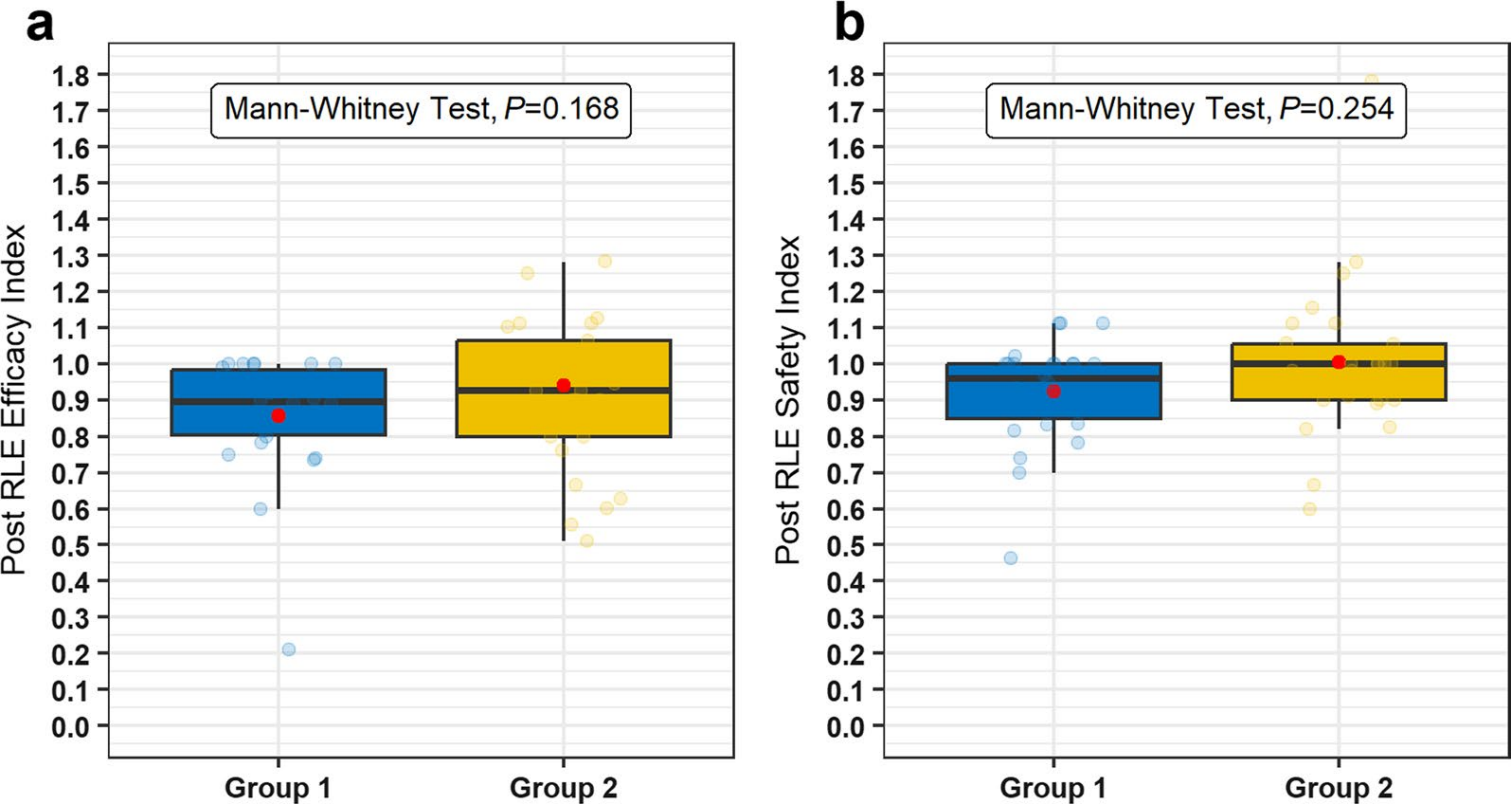
Efficacy and safety indices in patients undergoing RLE. **a** Boxplot showing efficacy index in groups 1 and 2 with no statistically significant differences between them (*P* = 0.168). **b** Boxplot showing safety index in groups 1 and 2 with no statistically significant differences (*P* = 0.254). RLE, refractive lens exchange

Table 3 displays preoperative and postoperative visual and refractive data, before and after lensectomy. Table 4 displays indicators of safety and efficacy in patients undergoing RLE. Post-lensectomy refractive results were slightly better in group 1 than in group 2 for MRSE, with a statistically significant difference (*P* < 0.001); however, no significant differences were observed in visual outcomes, namely UDVA (*P* = 0.647), CDVA (*P* = 0.343), UIVA (*P* = 0.059), UNVA (*P* = 0.382), binocular UIVA (*P* = 0.157), and binocular UNVA (*P* = 0.527) (Table 3). The post-RLE safety and efficacy indices in groups 1 and 2 were 0.96 and 1.00, and 0.89 and 0.93, respectively (*P* = 0.254 and 0.168; Table 4). The median (Q1, Q3) safety and efficacy index in patients undergoing RLE was 0.98 (0.90, 1.00) and 0.91 (0.80, 1.00), respectively; in cataract patients, these indices were 0.99 (0.90, 1.00) and 0.91 (0.76, 1.00), respectively. There was no difference in the above indices between the RLE and cataract groups (*P* = 0.729 and 0.548, Mann–Whitney test). Figure 1 and Table 4 show these indices for RLE eyes in both groups.

**Table 3:** Lensectomy data in group 1 and group 2

Parameter	Group 1 (n = 120)	Group 2 (n = 78)	*P* value
Pre-lensectomy			
* Sphere (D)*			
Range	− 5.00 to 4.00	− 2.75 to 4.25	
* Cylinder (D)*			
Range	− 2.25 to 0.00	− 2.00 to 0.00	
* MRSE (D)*			
Range	− 5.62 to 3.50	− 3.00 to 4.00	
Median (Q25/Q75)	1.00 (0.00/1.62)	1.25 (0.66/2.00)	**0.013**^**a**^
* J0 (D)*			
Range	− 0.94 to 0.98	− 0.94 to 0.70	
Median (Q25/Q75)	0.09 (− 0.25/0.26)	0.04 (− 0.21/0.29)	0.744^a^
* J45 (D)*			
Range	− 1.11 to 0.87	− 0.47 to 0.82	
Median (Q25/Q75)	− 0.09 (− 0.24/0.09)	0.00 (− 0.24/0.17)	0.128^a^
* UDVA (logMAR)*			
Range	0.00 to 1.70	0.00 to 1.30	
Mean (SD)	0.40 (± 0.35)	0.35 (± 0.23)	0.319^b^
* CDVA (logMAR)*			
Range	0.00 to 0.66	0.00 to 0.80	
Median (Q25/Q75)	0.08 (0.02/0.15)	0.05 (0.02/0.15)	0.488^a^
* UNVA bin (logMAR)*			
Range	0.18 to 1.00	0.18 to 1.00	
Mean (SD)	0.54 (± 0.30)	0.59 (± 0.29)	0.608^b^
* UIVA bin (logMAR)*			
Range	0.10 to 0.70	0.48 to 1.00	
Median (Q25/Q75)	0.30 (0.30/0.52)	0.90 (0.48/0.90)	0.052^a^
* UNVA (logMAR)*			
Range	0.10 to 1.00	0.18 to 1.00	
Mean (SD)	0.65 (± 0.24)	0.72 (± 0.26)	0.200^b^
* UIVA (logMAR)*			
Range	0.48 to 0.70	0.48 to 0.90	
Median (Q25/Q75)	0.48 (0.48/0.59)	0.90 (0.90/0.90)	0.102^a^
Lensectomy data			
* Time laser to lensectomy (years)*			
Range	1.64 to 17.01	6.97 to 20.53	
Mean (SD)	11.11 (± 3.49)	12.85 (± 3.18)	**< 0.001**^**b**^
* Axial length (mm)*			
Range	20.94 to 29.60	20.66 to 25.27	
Median (Q25/Q75)	22.63 (22.24/23.20)	22.51 (21.89/23.45)	0.073^a^
* IOL power (D)*			
Range	9.00 to 30.00	16.00 to 26.50	
Median (Q25/Q75)	22.00 (20.50/23.00)	22.50 (20.50/23.50)	0.377^a^
Post-lensectomy			
* Sphere (D)*			
Range	− 0.75 to 2.00	− 1.50 to 1.25	
* Cylinder (D)*			
Range	− 2.50 to 0.00	− 1.75 to 0.00	
* MRSE (D)*			
Range	− 0.75 to 1.00	− 1.62 to 1.00	
Mean (SD)	− 0.01 (± 0.38)	− 0.34 (± 0.51)	**< 0.001**^**b**^
* J0 (D)*			
Range	− 0.94 to 0.98	− 0.94 to 0.70	
Median (Q25/Q75)	0.09 (− 0.25/0.26)	0.04 (− 0.21/0.29)	0.744^a^
* J45 (D)*			
Range	− 1.11 to 0.87	− 0.47 to 0.82	
Median (Q25/Q75)	− 0.09 (− 0.24/0.09)	0.00 (− 0.24/0.17)	0.128^a^
* UDVA (logMAR)*			
Range	0.00 to 0.70	0.00 to 0.70	
Median (Q25/Q75)	0.10 (0.04/0.15)	0.07 (0.02/0.16)	0.647^a^
* CDVA (logMAR)*			
Range	0.00 to 0.40	0.00 to 0.30	
Median (Q25/Q75)	0.05 (0.01/0.12)	0.05 (0.01/0.10)	0.343^a^
* UNVA bin (logMAR)*			
Range	0.00 to 0.60	0.00 to 0.48	
Median (Q25/Q75)	0.10 (0.00/0.18)	0.10 (0.10/0.18)	0.527^a^
* UIVA bin (logMAR)*			
Range	0.00 to 0.48	0.10 to 0.60	
Median (Q25/Q75)	0.18 (0.18/0.30)	0.30 (0.18/0.30)	0.157^a^
* UNVA (logMAR)*			
Range	0.00 to 1.00	0.00 to 0.76	
Median (Q25/Q75)	0.18 (0.10/0.18)	0.18 (0.10/0.18)	0.382^a^
* UIVA (logMAR)*			
Range	0.00 to 0.48	0.18 to 0.60	
Median (Q25/Q75)	0.18 (0.18/0.30)	0.30 (0.18/0.30)	0.059^a^

*n* = number of available cases; *D* = diopters; *MRSE* = manifest refraction spherical equivalent; *UDVA* = uncorrected distance visual acuity; *SD* = standard deviation; *CDVA* = corrected distance visual acuity; *UIVA* = uncorrected intermediate visual acuity; *UNVA bin* = binocular uncorrected near visual acuity; *UIVA bin* = binocular uncorrected intermediate visual acuity; *UNVA* = uncorrected near visual acuity; *UIVA* = uncorrected intermediate visual acuity

^a^Mann–Whitney test

^b^*t*-test

Bold values indicate statistically significant

**Table 4:** Post-lensectomy safety and efficacy index in patients undergoing refractive lens exchange in group 1 and group 2

Parameter	Group 1	Group 2	*P* value
Safety index			
	0.96	1.00	0.254^a^
Range	0.46 to 1.11	0.60 to 1.78	
Median (Q25/Q75)	(0.85/1.00)	(0.90/1.06)	
Efficacy index			
	0.89	0.93	0.168^a^
Range	0.21 to 1.00	0.51 to 1.67	
Median (Q25/Q75)	(0.80/0.98)	(0.80/1.06)	

^a^Mann-Whitney test

**Fig. 2:**
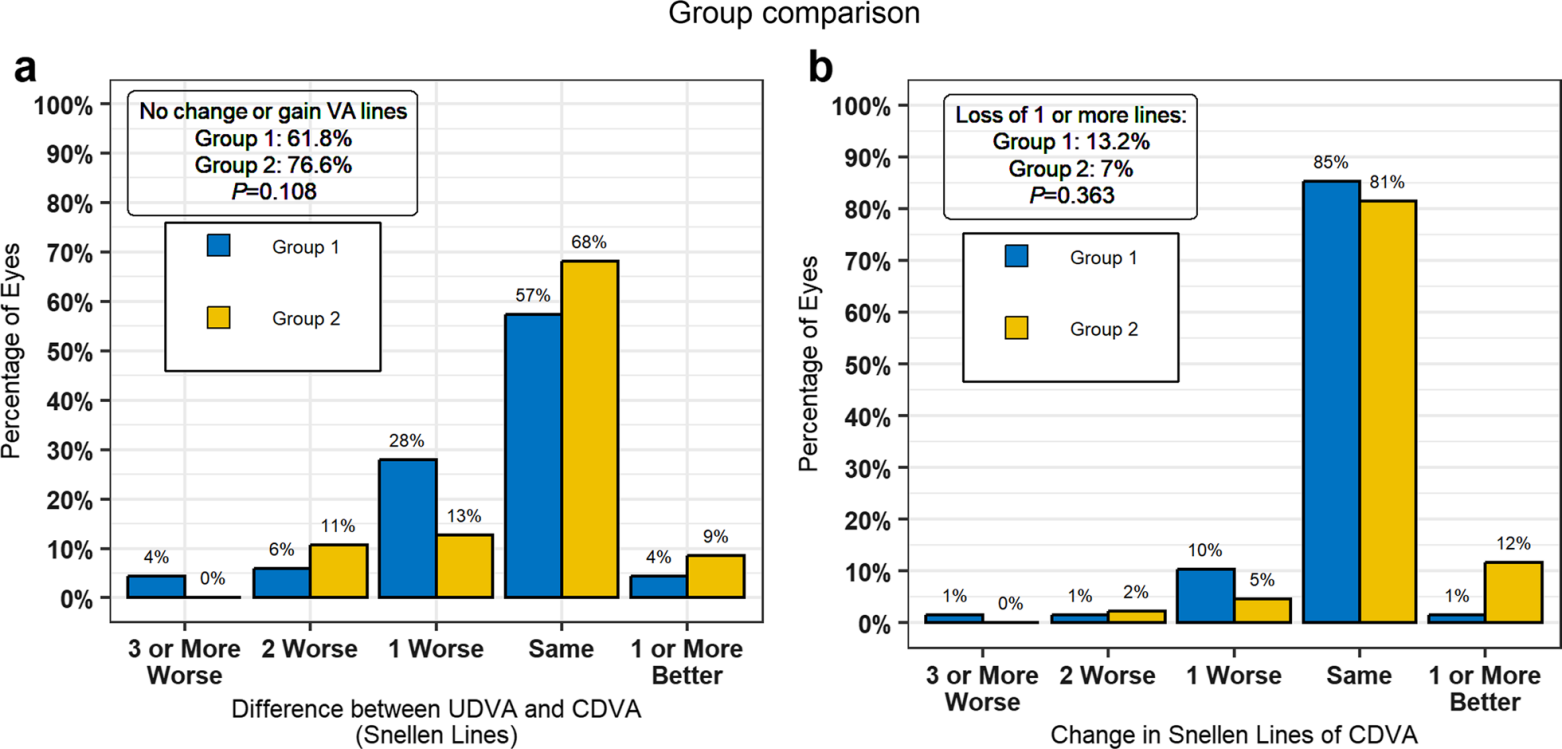
Percentages of eyes with no change, loss and gain of VA lines. **a** Efficacy of the procedure: difference between post-lensectomy UDVA and post-LCRS CDVA. **b** Safety of the procedure: change in Snellen lines of CDVA, risk of vision loss. VA, visual acuity; UDVA, uncorrected distance visual acuity; CDVA, corrected distance visual acuity; LCRS, laser corneal refractive surgery

In addition, Fig. 2 shows the efficacy in terms of the difference in Snellen lines between post-lensectomy UDVA and post-LCRS CDVA. No changes or improvements were observed in 61.8% of eyes in group 1 and 76.61% of eyes in group 2 (i.e., no statistically significant differences between the groups). Figure 2 also displays the standard safety graphics and risk of vision loss in both groups, with no statistical differences in the percentage of eyes that lost more than one line of CDVA (13.2% vs. 7.0% in groups 1 and 2, respectively). Post-lensectomy predictability was better in group 1, both for the ± 0.50 D and for the ± 1.00 D values (*P* = 0.017 and *P* = 0.002, respectively).

Subjective satisfaction data were analyzed only for patients with binocular implantation of the same trifocal IOL model, which was completed by 34 out of 46 patients (73.9%) in group 1 and 25 out of 33 patients (75.8%) in group 2. Visual acuity, dependence on spectacles/contact lenses, and satisfaction are presented in Tables 5, 6 and 7, respectively. Although the results were slightly better for Group 2, there were no significant differences between the groups for any of the issues studied.

**Table 5:** Patient satisfaction in terms of perceived outcomes for visual acuity

Parameter	Group 1 (n = 34)	Group 2 (n = 25)	Total (n = 59)	*P* value
Night vision				0.690
Good	31 (91.2%)	22 (88.0%)	53 (89.8%)	
Bad	3 (8.8%)	3 (12.0%)	6 (10.2%)	
Night driving				0.976
N-Miss	7	3	10	
Good	22 (81.5%)	18 (81.8%)	40 (81.6%)	
Bad	5 (18.5%)	4 (18.2%)	9 (18.4%)	
Near				0.697
Good	26 (76.5%)	18 (72.0%)	44 (74.6%)	
Bad	8 (23.5%)	7 (28.0%)	15 (25.4%)	
Intermediate				0.285
Good	28 (82.4%)	23 (92.0%)	51 (86.4%)	
Bad	6 (17.6%)	2 (8.0%)	8 (13.6%)	
Far				0.549
Good	28 (82.4%)	19 (76.0%)	47 (79.7%)	
Bad	6 (17.6%)	6 (24.0%)	12 (20.3%)	

All fisher’s exact test. N-Miss is the number of patients who did not respond to the questionnaire or to a particular item

**Table 6:** Patient satisfaction in terms of spectacle independence

Parameter	Group 1 (n = 34)	Group 2 (n = 25)	Total (n = 59)	*P* value
Near				0.157
N-Miss	0	1	1	
Independent	33 (97.1%)	21 (87.5%)	54 (93.1%)	
Dependent	1 (2.9%)	3 (12.5%)	4 (6.9%)	
Intermediate				
Independent	34 (100.0%)	25 (100.0%)	59 (100.0%)	
Dependent	0 (0.0%)	0 (0.0%)	0 (0.0%)	
Far				
Independent	34 (100.0%)	25 (100.0%)	59 (100.0%)	
Dependent	0 (0.0%)	0 (0.0%)	0 (0.0%)	

All fisher’s exact test. N-Miss is the number of patients who did not respond to the questionnaire or to a particular item

**Table 7:** Global satisfaction and percentage of patients that would or would not undergo the same treatment

Parameter	Group 1 (n = 34)	Group 2 (n = 25)	Total (n = 59)	*P* value
Satisfaction				0.730
N-Miss	1	0	1	
Satisfied	28 (84.8%)	22 (88.0%)	50 (86.2%)	
Not satisfied	5 (15.2%)	3 (12.0%)	8 (13.8%)	
Would you have surgery again?				0.911
Yes	31 (91.2%)	23 (92.0%)	54 (91.5%)	
No	3 (8.8%)	2 (8.0%)	5 (8.5%)	

All fisher’s exact test. N-Miss is the number of patients who did not respond to the questionnaire or to a particular item

## Discussion

Trifocal IOLs have proven effective in restoring useful uncorrected visual acuity at all distances after lens phacoemulsification surgery in eyes with cataract and/or presbyopia, and many studies have shown their ability to provide spectacle independence [2–4]. However, very few studies have evaluated their clinical results after LCRS [5–8, 11, 12]. To the best of our knowledge, no study has compared clinical performance considering the SA of the trifocal IOL and the previous LCRS procedure. Alfonso et al. studied refractive outcomes and visual quality after diffractive IOL implantation in eyes with previous hyperopic corneal ablation but with bifocal lenses [13, 14]. A trifocal IOL with neutral SA would provide better outcomes than a trifocal IOL with negative SA after hyperopic LCRS. Some authors have presented evidence regarding the improvement of depth of focus by manipulating SA [15]. Our purpose was to evaluate whether adding a more negative SA to an eye with an already reduced SA would fare better than not changing the SA. In our opinion, this is a paramount concern when attempting to maintain, improve, or at least not worsen visual outcomes by impairing corneal SA, because the need for retreatment and the aberrations brought about by hyperopic corneal ablation are more frequent than in the case of corneal myopic ablation [9, 10]. A limitation of the study is that UIVA and UNVA were analyzed but not the defocus curve of every patient; this could have given information about the distance-corrected intermediate and near visual acuity.

These two types of IOLs were compared previously by group after binocular implantation in 15 patients (30 eyes), although in eyes that had not been previously treated with LCRS [16]. Both models showed similar visual outcomes, with better refractive accuracy and less subjective visual disturbances in the RayOne group. Overall, our results showed better safety and efficacy in group 2, thus supporting the implantation of a trifocal IOL with neutral SA after hyperopic corneal ablation.

We were unable to identify published studies that analyzed whether a trifocal IOL with a neutral SA provided better visual and refractive outcomes and patient satisfaction than a trifocal IOL with a negative SA in eyes previously treated with hyperopic LCRS. Therefore, we were unable to draw comparisons with findings from similar studies. To the best of our knowledge, this study is the first to compare the visual and refractive outcomes of these two types of trifocal IOLs in eyes that underwent LCRS to treat hyperopia.

Our refractive results were significantly, albeit minimally, better in group 1 than in group 2, probably because our extensive surgical experience with the Physiol model enabled us to optimize the IOL constant and thereby obtain better refractive results.

The safety index was superior in group 2. Furthermore, even with no statistically significant differences, we found a loss of CDVA in both groups, although it was lower in group 2. Loss of lines of visual acuity of approximately 15%–20% and worse visual outcomes and safety have been reported after implantation of a negative SA trifocal IOL in eyes previously treated with LCRS for hyperopia [6–8]. In our study, we found a similar percentage of loss of lines in group 1, but almost half this value in group 2, supporting a possible key role of the SA of the IOL in the quality of vision after corneal refractive surgery. In our opinion, these findings are crucial because the loss of CDVA lines could be related to the amount of SA induced in the eye and to the implanted IOL. This is not surprising because an IOL with a negative SA increases the negative SA induced by prior hyperopic corneal ablation. Our group recently found poorer visual outcomes in eyes with a higher previous grade of hyperopic LCRS and more negative Z4(0) values when these eyes received a trifocal IOL with a negative SA [17]. However, the outcomes could be influenced not only by the SA of the IOL, but also other issues such as the design of the IOL. In fact, the difference in SA of both IOLs is small and visual satisfaction was similar; differences could appear if the difference of SA between the IOLs were higher. Further research comparing other models of trifocal IOLs with different SA could show more evidence in patients with previous corneal laser refractive surgery.

Patient satisfaction was good. Vision scores, spectacle/contact lens independence, and the percentage of patients who would repeat the procedure were, as expected, lower than those in other studies after trifocal IOL implantation with no previous LCRS procedure [4].

The aim of the study was not to establish a relationship between the aberration of the implanted lens and the corneal aberrations induced by the previous hypermetropic ablation. Rather, it was to determine whether the aberration of the implanted lens has any impact on visual and refractive outcomes in line with the previous hypothesis that a lens with neutral aberration is more suitable after hypermetropic corneal ablation reducing the risk of loss of CDVA lines. Therefore, we are aware of the need for further studies including measurements such as aberrometry, defocus curves and contrast sensitivity to confirm our results and to clarify whether our findings are related to residual refraction or to higher order aberrations induced by each IOL.

Our study is limited by its retrospective design and the absence of quality of vision parameters. For instance, UIVA and UNVA were analyzed, but not the defocus curve for each patient, which could have provided information on distance-corrected near and intermediate visual acuity. However, we performed a real-life subjective patient satisfaction survey and assessed spectacle independence using the same questionnaire applied in previous studies by our group [4, 6, 7]. In addition, the large number of surgeons, different excimer lasers and measurement devices used during the study period constituted an unavoidable drawback of this retrospective multicenter study. Nevertheless, our group followed mandatory medical and surgical protocols at all our centers. Furthermore, our study is the first to assess whether an aspherical trifocal IOL with a neutral SA provides better visual and refractive outcomes and patient satisfaction than an aspherical trifocal IOL with a negative SA in eyes that had previously undergone hyperopic LCRS.

## Conclusion

In eyes previously treated with hyperopic corneal ablation, implantation of a trifocal IOL with neutral SA provided better efficacy and safety outcomes but worse predictability outcomes than those obtained with a trifocal model with negative SA.

## Data Availability

The authors confirm that the data supporting the conclusions of this article are included within the article and its additional files.

## Abbreviations

CDVA: Corrected distance visual acuity
IOL: Intraocular lens
LASIK: Laser-assisted in situ keratomileusis
LCRS: Laser corneal refractive surgery
MRSE: Manifest refraction spherical equivalent
RLE: Refractive lens exchange
SA: Spherical aberration
UDVA: Uncorrected distance visual acuity
UIVA: Uncorrected intermediate visual acuity
UNVA: Uncorrected near visual acuity
